# Characteristics and predictors of persistent somatic symptoms in patients with cardiac disease

**DOI:** 10.1038/s41598-024-76554-z

**Published:** 2024-10-26

**Authors:** Caroline Clifford, Bernd Löwe, Sebastian Kohlmann

**Affiliations:** 1 Department of Psychosomatic Medicine and Psychotherapy, University Medical Center Hamburg-Eppendorf, Martinistraße 52, Building W 37, Room 6010 a, 20246 Hamburg, Germany; 2 Department of General Internal and Psychosomatic Medicine, University Medical Center Heidelberg, Heidelberg, Germany

**Keywords:** Cardiac disease, Cluster analysis, Depression, Healthcare utilization, Somatic Symptom Scale-8, Persistent somatic symptoms, Cardiology, Health care, Medical research, Risk factors, Signs and symptoms

## Abstract

Persistent somatic symptoms (PSS) are a diagnostic core criterion of the somatic symptom disorder. This longitudinal study aims to determine the frequency of PSS in patients with cardiac disease, identify potential predictive factors, and investigate its impact on healthcare utilization. Somatic symptoms were assessed with the Somatic Symptom Scale-8 four times over the course of three months in consecutively approached cardiac outpatients. Patients were grouped having PSS vs. not having PSS following a psychometric-driven approach based on the SSS-8 cut-off score and a data-driven approach applying cluster analysis. T-tests were performed to compare the characteristics between patients having vs. not having PSS. To identify predictors of group affiliation, we conducted multivariable logistic regressions. Additionally, analyses of covariance were used to further examine associations between healthcare utilization and group affiliation. The study included 95 patients (30.5% female) with a mean age of 60.5 years (*SD* = 8.7). All patients had at least one of the following cardiac diseases recorded in their medical history: coronary heart disease (*n* = 51), myocardial infarction (*n* = 21), valve disease (*n* = 22), cardiomyopathy (*n* = 15), cardiac dysrhythmia (*n* = 43), and heart failure (*n* = 12). 30 (32%) were grouped having PSS according to the psychometric-driven approach and 27 (28%) according to the data-driven approach. For both approaches, patients with PSS were more likely to be female, unemployed, reporting angina pectoris, having higher depression, and higher anxiety severity (for all: *p* ≤ 0.05). Predictors of PSS group affiliation were female gender, higher age, depression severity, and angina pectoris (for all: *p* ≤ 0.015). Patients with PSS more frequently visited general practitioners and cardiologists compared to patients without PSS (*p* ≤ 0.013). Enhancing our knowledge of PSS in patients with cardiac disease could help to improve identification of patients’ specific needs and the factors to consider in diagnosis and individualized treatment.

**Supplementary Information:**

The online version contains supplementary material available at 10.1038/s41598-024-76554-z.

## Introduction

Approximately 23% of patients visiting general practitioners in Germany are affected by somatic symptom disorder (SSD)^1^. One of the diagnostic criteria for somatic symptom disorder, as outlined in the DSM-5, is the persistence of somatic symptoms^2^. Identifying SSD in individuals with cardiac disease poses a particular challenge due to the high diversity of symptoms and the overlap of phenomenology with SSD. Given the lack of evidence regarding persistent somatic symptoms (PSS) in patients with cardiac diseases, the objective of this longitudinal study is to approach a definition, approximate the prevalence, and examine the associated characteristics of PSS.

The prevalence of subjective somatic symptoms in patients with cardiac disease is high and comparable to those of patients with cancer^3,4^. Independent of cardiac disease markers, subjective somatic symptoms are associated with death and hospitalization^5–7^. From other somatic diseases, it is well known that, in particular, the persistence of somatic symptoms carries far-reaching consequences such as a more severe course of the disease, increased need of clinical treatment, and increased mortality risk^8–10^. Persistent somatic symptoms (PSS) are defined as “subjectively distressing somatic complaints, irrespective of their etiology, that are present on most days for at least several months”^10^. Löwe et al.^10^ described the operationalization of PSS through somatic symptom severity experienced by the patient. PSS predict functional impairment, disability, decreased quality of life, and a higher probability of developing affective disorders^3,11,12^.

Somatic symptoms result from a complex interplay of biological, psychological, and social processes^13^. According to the model by Löwe et al., which describes risk factors and mechanisms for somatic symptom persistence, sociodemographic, psychosocial, and biomedical factors play a key role in developing short-term somatic symptoms^10^. Other psychosocial and biomedical factors such as cognitive-perceptual and emotional mechanisms or disease-specific factors as well as personal expectations, increase the chances of developing PSS^10^. According to the model, it could be valuable to understand the development of PSS in patients with cardiac disease. So far, the model has already been applied to other somatic diseases such as chronic kidney disease, primary biliary cholangitis, irritable bowel syndrome, and ulcerative colitis^14–16^.

From a healthcare perspective, it also appears essential to examine PSS in patients with cardiac disease: Unsatisfactory encounters with the healthcare system and adverse treatment experienced by patients with PSS often result in unnecessary, frequent, and potentially harmful overuse of healthcare which engenders substantial costs^17–19^. It is understood that individuals with PSS commonly seek assistance from their general practitioners^20^. Further research into the actual healthcare situation of patients with cardiac disease and PSS is warranted to facilitate early identification and the development of patient-oriented interventions^18,21^.

To date, predicting the occurrence of PSS in patients with cardiac disease remains a challenge. By gaining insight into the predictors for group affiliation, specifically identifying which patients develop PSS and which do not, we can infer the underlying factors and needs of this patient group. Research has so far focused on the consequences of PSS in different patient groups such as increased mortality and higher probability of developing affective disorders^3,10^. This study will focus on the characteristics and predictors of patients with PSS.

The objective of this longitudinal study is to determine PSS in patients with cardiac disease over a time period of three months. In addition to approximating a definition of PSS in this longitudinal study, the aim is also to approximate the prevalence of PSS in patients with cardiac disease. Associations of psychological factors, as well as cardiac characteristics and comorbidities with somatic symptoms, are tested as predictors for PSS. The following research questions are examined exploratively:

1. Which biomedical and psychological factors characterize patients with cardiac disease who report persistent somatic symptoms?
2. Which biomedical and psychological factors predict persistence of somatic symptoms over the course of three months in patients with cardiac disease?
3. Do patients with cardiac disease with persistent somatic symptoms report more healthcare utilization compared to patients without persistent somatic symptoms?

## Materials and methods

### Design

Outpatients from the University Heart Center and from a non-university cardiac outpatient clinic in Hamburg (Germany) who had a regular consultation appointment were approached by telephone. Patients with a cardiac disease according to medical records, or with at least two risk factors for developing coronary heart disease were contacted. Data from patients was included in the analyses if a cardiac disease had been confirmed during consultation. Further inclusion criteria were 18 years or older, access to a telephone, and sufficient language skills (German). Exclusion criteria were life-threatening health status, a severe somatic or psychiatric disorder requiring urgent treatment, severe cognitive, motoric, or visual difficulties, hospitalization within the previous week, a surgical procedure with at least a three-day hospitalization within the last two months, myocardial infarction within the last three months, any skeletal disease or no written informed consent.

The longitudinal study consisted of four assessments: Two weeks before a regular cardiac consultation, the first data assessment took place via telephone (T0). On the day of the cardiac consultation (T1), the second data assessment was conducted in person. One month (T2) and three months (T3) after consultation, further assessments were conducted by telephone. At each timepoint the following data was collected: the Somatic Symptom Scale (SSS-8), the Patient Health Questionnaire (PHQ-9), the Generalized Anxiety Disorder Scale (GAD-7), and the Canadian Cardiovascular Society (CCS) class. Sociodemographic data was gathered at baseline, while healthcare utilization was assessed at three months after baseline assessment by asking the patients about their visits to a general practitioner and cardiologist over the previous three months. Biomedical factors, including the number of cardiac diseases, were obtained from medical records and had to be confirmed during the cardiac consultation. In the psychometric- and data-driven approach to identify patients with PSS, we included the SSS-8 sum scores from two weeks to three months after baseline assessment (T1 to T3). Data was assessed as part of the Risk Act study (Clinicaltrials.gov identifier: NCT02802254). The study was approved by the Ethics Committee of the Medical Chamber, Hamburg, Germany (PV5199). All methods were carried out in accordance with relevant guidelines and regulations.

### Measurements

#### Sociodemographic data

Patients completed questionnaires assessing sociodemographic data, including age, gender, language, living situation, education, and employment.

#### Somatic symptom severity

Somatic symptom severity was measured with the Somatic Symptom Scale (SSS-8), which assesses the presence and severity of common somatic symptoms (e.g. back pain, stomach or bowel problems, chest pain, and fatigue)^22^. The SSS-8 is brief, valid, and reliable (*alpha* = 0.81). A cut-off score for identifying somatic symptom burden is set at 4 points^22^. This cut-off score has been validated in cross-sectional studies; there is no empirical cut-off score to define the persistence of somatic symptoms.

#### Biomedical factors

To measure biomedical factors, the number of cardiac diseases was assessed. Therefore, medical records were screened and the diagnoses had to be confirmed during the cardiac consultation including coronary heart disease, myocardial infarction, valve disease, cardiomyopathy, cardiac dysrhythmia, and heart failure. Furthermore, the number of comorbidities and medications were obtained from medical records. Comorbidities were defined as non-cardiac diagnoses such as liver or kidney diseases, cancer, epilepsy, etc., but also included mental illnesses such as depression, anxiety disorder, or addiction. Additionally, cardiac risk factors such as hypertension, diabetes, hyperlipidemia, smoking, obesity, and positive family history were assessed through self-report.

#### Cardiac factors

The impairment caused by angina pectoris was rated according to the Canadian Cardiovascular Society (CCS) class^23^. This classification system is an established marker of the functional severity of heart diseases^24^. According to the impairment level, four classes are determined, ranging from ‘no impairment at all’ to ‘impairment even at resting’. As symptom impairment increases, the classes are rated higher.

#### Psychological factors

The Patient Health Questionnaire (PHQ-9) was used to measure depression severity. The PHQ-9 assesses how often patients have experienced the nine most common depressive symptoms over the previous two weeks. The score range is 0–27 points. Cut-off scores are 5 (mild depression), 10 (moderate depression), and 15 points (severe depression). The PHQ-9 is a valid and reliable instrument^25,26^.

The Generalized Anxiety Disorder Scale (GAD-7) was used to evaluate the severity of anxiety over the last two weeks. The score range is 0–21 points. Cut-off scores are 5 (mild anxiety), 10 (moderate anxiety), and 15 points (severe anxiety). The GAD-7 has valid and reliable case-finding properties for the most common anxiety disorders^27,28^.

#### Healthcare utilization

To quantify healthcare utilization, patients were asked at the last follow-up how often they visited a general practitioner and a cardiologist during the previous three months.

### Statistical analysis

To define PSS in patients with cardiac disease, two methodological approaches were applied: (a) psychometric-driven approach, based on the cut-off of the SSS-8, and (b) data-driven approach, applying a cluster analysis on the SSS-8 sum scores. By applying two approaches, we aimed to cross-validate our findings concerning PSS. According to the psychometric-driven approach, patients were grouped as having PSS if they scored four points or more on the SSS-8 at all assessment points except for the baseline measurement (PSS group vs. no PSS group). The predictive variables were derived from the baseline data, which is why the baseline measurement of the SSS-8 was not considered in developing the group affiliations. Applying the data-driven approach, patients were grouped according to the results of a cluster analysis (PSS cluster vs. no PSS cluster). Given the exploratory nature of our research question, we opted for a hierarchical cluster analysis using the Ward method, without pre-specifying the number of clusters to be identified. The cluster analysis was conducted using the SSS-8 sum scores for each patient, incorporating data from two weeks to three months after baseline assessments (T1 to T3). Data from the baseline assessment (T0) of the SSS-8 was not included in the cluster analysis, as variables from this time point were used as predictors. Based on the dendrogram and the explained variance observed in the cluster analysis, we identified a two-cluster solution as meaningful regarding the presence of PSS in our population of patients with cardiac disease (see Supplement A and B).

Firstly, descriptive analyses as well as t-tests and chi-squared tests were conducted to identify sociodemographic, biomedical, and psychological characteristics that differentiate between patients with and without PSS longitudinally according to the psychometric- and data-driven approach. Analyses were conducted for both approaches and group affiliation was treated as independent variable in each case.

Secondly, multivariable logistic regression was performed to identify predictors of group affiliation (PSS vs. no PSS). Concerning the biomedical factors, the following predictors were entered into the model: the number of cardiac diseases, cardiac risk factors, comorbidities, and medication, as well as angina pectoris defined by the CCS class. As psychological factors depression severity (PHQ-9) and anxiety severity (GAD-7) were entered. To understand which factors contributed to predicting PSS, we used group affiliation based on the SSS-8 sum scores as the dependent variable in the logistic regression. The model was adjusted for age and gender. Two models, according to the psychometric-driven and the data-driven approach, were analyzed.

As a third step, correlations and analyses of covariance (ANCOVA) were performed to test the association between PSS group affiliation (predictor) and healthcare utilization (outcome). Direct maximum likelihood estimation was applied to handle missing follow-up data. Missings were at random, ranging between one and fifteen percent. Two-tailed p-values < 0.05 were considered significant. We used SPSS 27 for statistical analyses. Multicollinearity and further statistical prerequisites were tested. As a sensitivity analysis, the groups of patients with and without PSS were formed, excluding three items from the SSS-8 which are related to cardiac disease (chest pain or shortness of breath, dizziness, feeling tired or having low energy)^3^. Excluding these items did not show differences in the results. The data that supports the findings of this study is available on request from the corresponding author.

## Results

### Sample

In total, 270 outpatients having a regular cardiac consultation were approached via telephone and were asked to participate in a regular data assessment over a three-month time period. Of those, 95 were eligible and gave informed consent. Others were excluded due to missing informed consent (*n* = 120), language difficulties (*n* = 18), missing contact details (*n* = 9), severe somatic disease (*n* = 9), skeletal disease (*n* = 9), no cardiac disease (*n* = 6), an operation within the last two months (*n* = 2), a myocardial infarction within the last three months (*n* = 1) or a life-threatening status (*n* = 1). There was no drop-out during the longitudinal study and all 95 patients participated in the three-month assessment. As the data derives as a secondary analysis, we conducted a post-hoc power analysis to determine the statistical power. Given a sample size of *n* = 95, our analyses showed a power of 1 −*β* = 0.70 to detect medium-sized effects (*R^2* = 0.13) on identifying groups (with PSS vs. without PSS) when testing nine predictors in logistic regression models^29^. Given a sample size of *n* = 95, our analyses showed a power of 1 −*β* = 0.67 to detect medium-sized effects (*f* = 0.25) in the conducted ANCOVAs. Regarding the recommended sample size for conducting cluster analysis, research suggests that traditional assumptions about statistical power only apply partially. However, a range of 20–30 observations per expected subgroup is generally advised to ensure meaningful and stable results^30^.

According to the psychometric-driven approach, *n* = 30 (32%) of the patients had 4 points or above on the SSS-8 at all assessment points included. According to the data-driven definition of PSS, *n* = 27 (28%) of the patients were assigned to the PSS cluster. The overlap concerning group membership between both approaches was 95%.

Table 1 summarizes the characteristics of the sample for all patients and according to the psychometric-driven and data-driven approach for PSS definitions.

**Table 1:** Baseline characteristics of *n* = 95 patients with cardiac disease.

Characteristics	All patients	Groups based on psychometric-driven approach			Groups based on data-driven approach		
	(*n* = 95)	PSS group	No PSS group	*p* value	PSS cluster	No PSS cluster	*p* value
		(*n* = 30)	(*n* = 65)		(*n* = 27)	(*n* = 68)	
Sociodemographics, n (%)							
Age, mean (SD), years	60.5 (8.7)	60.2 (8.9)	60.7 (8.6)	0.798	59.1 (8.7)	61.1 (8.6)	0.327
Gender, female	29 (30.5)	17 (56.7)	12 (18.5)	< 0.001	16 (59.3)	13 (19.1)	< 0.001
German mother tongue	90 (94.7)	29 (96.7)	61 (93.8)	0.567	27 (100.0)	63 (92.6)	0.148
Living alone	15 (15.8)	6 (20.0)	9 (13.8)	0.445	5 (18.5)	10 (14.7)	0.646
≥ 10 years of formal education	62 (65.3)	20 (66.7)	42 (64.6)	0.845	19 (70.4)	43 (63.2)	0.510
Employed	37 (38.9)	7 (23.3)	30 (46.2)	0.034	6 (22.2)	31 (45.6)	0.035
Clinical characteristics, n (%)							
Coronary heart disease	51 (53.7)	12 (40.0)	39 (60.0)	0.069	10 (37.0)	41 (60.3)	0.040
Myocardial infarction	21 (22.1)	7 (23.3)	14 (21.5)	0.845	6 (22.2)	15 (22.1)	0.986
Valve disease	22 (23.2)	10 (33.3)	12 (18.5)	0.110	7 (25.9)	15 (22.1)	0.687
Cardiomyopathy	15 (15.8)	5 (16.7)	10 (15.4)	0.873	5 (18.5)	10 (14.7)	0.646
Cardiac dysrhythmia	43 (45.3)	16 (53.3)	27 (41.5)	0.283	15 (55.6)	28 (41.2)	0.204
Heart failure	12 (12.6)	6 (20.0)	6 (9.2)	0.142	6 (22.2)	6 (8.8)	0.076
Number of cardiac diseases, mean (SD)	2.1 (1.3)	2.5 (1.6)	2.0 (1.1)	0.125	2.4 (1.6)	2.0 (1.2)	0.246
Number of comorbidities, mean (SD)	2.5 (1.5)	2.5 (1.5)	2.6 (1.6)	0.775	2.5 (1.5)	2.5 (1.6)	0.897
Number of medication, mean (SD)	5.2 (2.7)	6.0 (2.8)	4.9 (2.6)	0.066	5.8 (2.7)	5.0 (2.7)	0.170
Somatic symptoms, mean (SD)							
Somatic symptom severity [SSS-8]	6.5 (5.2)	11.6 (4.6)	4.2 (3.6)	< 0.001	12.7 (4.0)	4.1 (3.3)	< 0.001
Stomach or bowel problems	0.5 (1.0)	1.1 (1.5)	0.2 (0.5)	0.002	1.1 (1.5)	0.2 (0.6)	0.005
Back pain	1.2 (1.2)	1.9 (1.3)	0.8 (1.0)	< 0.001	2.2 (1.3)	0.8 (1.0)	< 0.001
Pain in the arms, legs, or joints	1.1 (1.1)	1.8 (1.2)	0.8 (0.9)	< 0.001	2.0 (1.0)	0.7 (1.0)	< 0.001
Headaches	0.5 (1.0)	0.7 (1.1)	0.4 (0.9)	0.255	0.7 (1.1)	0.4 (0.9)	0.164
Chest pain or shortness of breath	0.9 (1.1)	1.8 (1.1)	0.6 (0.9)	< 0.001	1.8 (1.0)	0.6 (1.0)	< 0.001
Dizziness	0.4 (0.8)	1.0 (1.1)	0.2 (0.5)	0.001	1.0 (1.1)	0.2 (0.5)	0.001
Feeling tired or having low energy	1.0 (1.1)	1.7 (1.1)	0.7 (0.9)	< 0.001	1.9 (1.2)	0.6 (0.8)	< 0.001
Trouble sleeping	0.9 (1.2)	1.6 (1.4)	0.5 (0.9)	< 0.001	1.9 (1.4)	0.5 (0.8)	< 0.001
Cardiac risk factors, n (%)							
Hypertension	68 (71.6)	22 (73.3)	46 (70.8)	0.797	19 (70.4)	49 (72.1)	0.869
Diabetes	21 (22.1)	5 (16.7)	16 (24.6)	0.385	2 (7.4)	19 (27.9)	0.030
Hyperlipidemia	43 (45.3)	16 (53.3)	27 (41.5)	0.283	15 (55.6)	28 (41.2)	0.204
Smoking	30 (31.6)	10 (33.3)	20 (30.8)	0.803	10 (37.0)	20 (29.4)	0.471
Obesity	44 (46.3)	16 (53.3)	28 (43.1)	0.351	13 (48.1)	31 (45.6)	0.821
Family history	32 (33.7)	9 (30.0)	23 (35.4)	0.606	8 (29.6)	24 (35.3)	0.598
Number of risk factors, mean (SD)	2.5 (1.1)	2.6 (0.9)	2.5 (1.2)	0.571	2.5 (0.9)	2.5 (1.2)	
Angina pectoris, n (%)							
CCS Class 1	67 (70.5)	11 (36.7)	56 (86.2)	< 0.001	9 (33.3)	58 (85.3)	< 0.001
CCS Class 2	11 (11.6)	6 (20.0)	5 (7.7)	0.081	5 (18.5)	6 (8.8)	0.183
CCS Class 3	6 (6.3)	5 (16.7)	1 (1.5)	0.005	5 (18.5)	1 (1.5)	0.002
CCS Class 4	11 (11.6)	8 (26.7)	3 (4.6)	0.002	8 (29.6)	3 (4.4)	0.001
Psychological factors, mean (SD)							
Depression severity [PHQ-9]	4.5 (4.5)	7.5 (5.1)	3.1 (3.3)	< 0.001	8.4 (5.2)	2.9 (3.0)	< 0.001
Anxiety severity [GAD-7]	2.9 (3.4)	4.5 (3.5)	2.2 (3.2)	0.003	5.1 (3.9)	2.1 (2.9)	0.001

SSS-8 = Somatic Symptom Scale-8; CCS = Canadian Cardiovascular Society; PHQ-9 = Patient Health Questionnaire-9. GAD-7 = Generalized Anxiety Disorder Scale-7; SD = Standard deviation.

In addition, we took a closer look at the distribution of the somatic symptom severity according to the Somatic Symptom Scale (SSS-8) in the sample for all patients and according to the psychometric-driven and data-driven approach for PSS definition. The scale for each item stretches from 0 (“not at all”) to 4 (“very much”) describing how much the patient has been disturbed by the somatic symptom during the previous seven days. The average sum score calculated through the eight items for all patients is 6.5 points (*SD* = 5.2 points). For patients with PSS, the sum score is between 11.6 points (*SD* = 4.6 points) for the psychometric-driven approach and 12.7 points (*SD* = 4.0 points) for the data-driven approach. For patients without PSS, the sum score is between 4.1 points (*SD* = 3.3 points) and 4.2 points (*SD* = 3.6 points). The results show a higher symptom severity in patients with PSS for every assessed symptom except headache. Further details can be seen in Table 1.

### Biomedical and psychological characteristics of patients with and without PSS

Patients with and without PSS differed at baseline concerning gender, employment status, angina pectoris (CCS class), somatic symptom severity, and psychological factors. Patients with PSS were more likely to be female, unemployed, to suffer from more severe angina pectoris, to describe higher somatic symptom severity, and to show higher depression and anxiety scores (for all tests; *p* < 0.005). These differences could be found both in the psychometric-driven and data-driven grouping approach. Groups did not differ significantly regarding age, further sociodemographic information, and clinical characteristics such as cardiac diseases, comorbidities, medication, and cardiac risk factors. Further details can be seen in Table 1.

### Biomedical and psychological factors as predictors of PSS

**Fig. 1:**
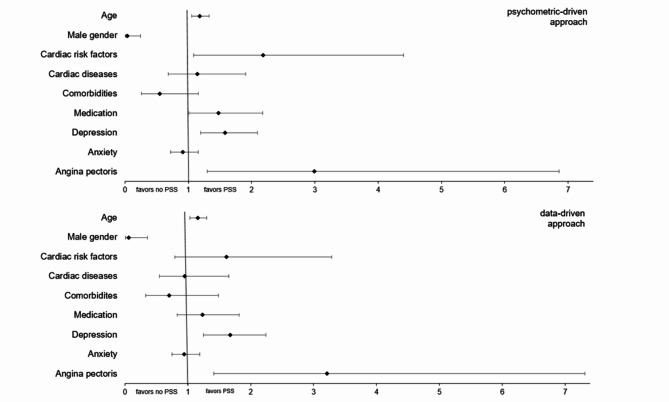
Biomedical, psychological, and sociodemographic predictors for the occurrence of persistent somatic symptoms in patients with cardiac disease based on both the psychometric- and the data-driven approach.

To visualize the differential effects of biomedical and psychological factors in patients with PSS, the unstandardized regression coefficients and confidence intervals are displayed in Fig. 1. Predictors entered in the model were age, gender, cardiac risk factors, number of cardiac diseases, comorbidities, and medication as well as depression, anxiety, and angina pectoris scores. To predict group membership for PSS for patients according to the psychometric-driven approach, the regression model showed good prediction abilities (*-2 Log-Likelihood* = 54.9; *Cox & Snell R^2* = 0.488; *Nagelkerkes R^2* = 0.685). Positive prediction abilities were also seen in the prediction of PSS for patients according to the data-driven approach (*-2 Log-Likelihood* = 49.2; *Cox & Snell R^2* = 0.491; *Nagelkerkes R^2* = 0.705). Stable predictors for PSS according to the psychometric-driven approach were age (*OR* = 1.18; *p* = 0.005), gender (*OR* = 0.04; *p* = 0.001), PHQ-9 depression score (*OR* = 1.58; *p* = 0.001) and CCS class indicating the severity of angina pectoris (*OR* = 2.99; *p* = 0.010). Stable predictors for PSS according to the data-driven approach were similar: age (*OR* = 1.16; *p* = 0.015), gender (*OR* = 0.06; *p* = 0.002), PHQ-9 depression score (*OR* = 1.68; *p* = 0.001) and CCS class indicating the severity of angina pectoris (*OR* = 3.21; *p* = 0.005). To adjust for multiple comparisons, the significance level was interpreted according to the Benjamini Hochberg formula^31^.

### Association of persistent somatic symptoms with healthcare utilization

**Fig. 2:**
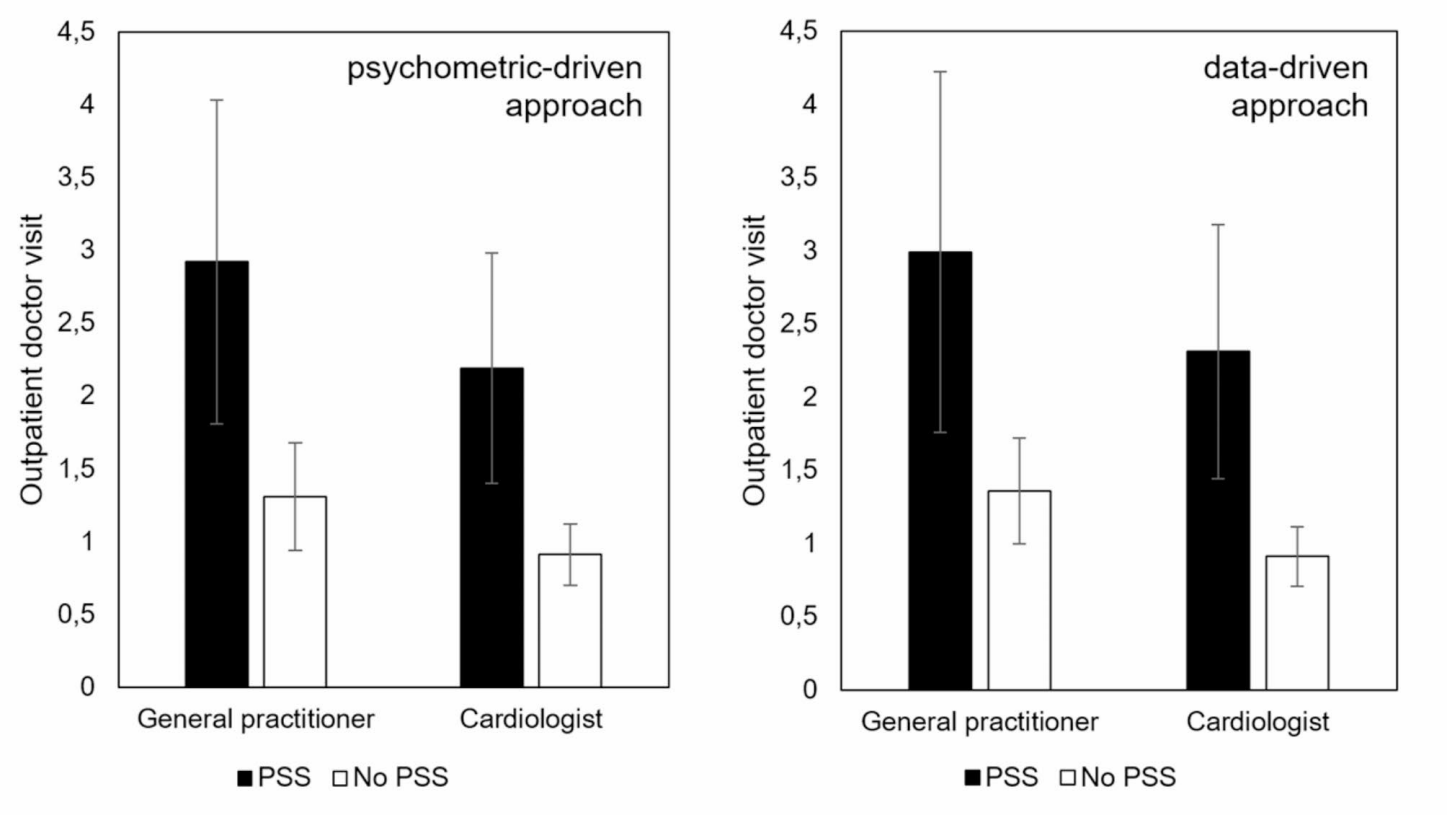
Healthcare utilization comparison of patients with cardiac disease with and without persistent somatic symptoms according to the psychometric-driven and data-driven approach. The total number of outpatient visits to a general practitioner and to a cardiologist are compared.

As can be seen in Fig. 2, the groups with and without PSS according to the psychometric-driven and data-driven approach, were compared regarding outpatient visits.

Based on the psychometric-driven approach, patients with PSS showed a mean of 2.9 (*SD* = 3.0) visits to a general practitioner within a three-month period compared with 1.3 (*SD* = 1.5) visits on average for patients without PSS (*p* = 0.008). Patients with PSS showed a mean of 2.2 (*SD* = 2.1) visits to a cardiologist within a three-month period compared with 0.9 (*SD* = 0.8) visits on average for patients without PSS (*p* = 0.003). The ANCOVA adjusted for age and gender showed differences between patients with and without PSS for visits to the general practitioner (*F* = 7.22; *p* = 0.009) and the cardiologist (*F* = 12.9; *p* = 0.001).

Based on the data-driven approach, patients with PSS showed a mean of 3.0 (*SD* = 3.1) visits to a general practitioner within a three-month period compared with 1.4 (*SD* = 1.5) visits on average for patients without PSS (*p* = 0.013). Patients with PSS showed a mean of 2.3 (*SD* = 2.2) visits to a cardiologist within a three-month period compared with 0.9 (SD = 0.8) visits on average for patients without PSS (*p* = 0.003). The ANCOVA adjusting for age and gender showed significant differences between patients with and without PSS for visits to the general practitioner (*F* = 6.53; *p* = 0.012) and the cardiologist (*F* = 14.45; *p* < 0.001).

## Discussion and conclusion

### Discussion

To the best of our knowledge, this is the first longitudinal study identifying characteristics and predictors of persistent somatic symptoms (PSS) in patients with cardiac disease. Over the course of three months, one-third of the study population showed PSS. The psychometric- and the data-driven approach, revealed similar rates of PSS. Patients with cardiac disease showing PSS were more likely to be female, to be unemployed, and to suffer from more severe angina pectoris. Additionally, they also showed higher depression and anxiety severity. Higher age, female gender, more severe angina pectoris, and higher depression severity were significant and stable predictors for PSS over the course of three months. Biomedical factors such as the number of cardiac diseases, comorbidities, cardiac risk factors, or number of medications could not be identified as predictors for PSS. Both the psychometric-driven as well as the data-driven approach indicate that patients with PSS showed a higher number of outpatient visits.

These results enabled us to gain more differentiated insights into which factors from the bio-psycho-social model play a role, especially for patients with cardiac disease^10^. Concerning biomedical factors angina pectoris seems to be relevant while somatic comorbidities, cardiac risk factors, and the number of medications do not play a major role in predicting PSS. Depression, as a psychological factor predicting PSS, appears to be a risk factor in cardiac disease while anxiety does not seem to predict PSS. The results underline the importance of an integrated assessment of biomedical and psychological factors for patients with cardiac disease. Future research should examine how psychological needs in addition to biomedical examinations can be included in diagnostics and patient-oriented treatment to reduce symptom severity and persistence as well as improve the quality of life of patients with cardiac disease.

The main focus of this longitudinal study was to gain knowledge about which patients with cardiac disease develop PSS and which characteristics and predictors play a role. DeVon et al. (2017) published a systematic review examining symptom profiles in individuals with cardiac disease^32^. The findings revealed variability in both the number and structure of identified profiles. While some studies classified symptoms based on severity, others emphasized symptom quality. Additionally, the identified profiles often included both somatic and psychological factors across different studies^32^. Riegel et al. (2010) provided one example of symptom profiles for patients with cardiovascular disease^33^. They identified four clusters (classic acute coronary syndrome, pain symptoms, stress symptoms, and diffuse symptoms) and were able to find correlations between symptom profile and mortality. Further examples of identified symptom profiles in individuals with cardiac disease include the six distinct symptom clusters found by Hu et al. (2020): fatigue, dyspneic, discomfort, congestive, ischemic, and emotional clusters^34^. Denfeld et al. (2020) identified profiles such as congruent-mild, incongruent, and congruent-severe, also including both physical and affective heart failure symptoms^35^. As yet there is no knowledge about the persistence within these symptom profiles. In future research increased differentiated knowledge can be gained by forming symptom profiles and analyzing these regarding persistence. Additionally, it could be of interest to focus on patients at risk prior to developing a cardiac disease. This could be helpful for the early identification and may be used to develop individualized interventions such as screening or interdisciplinary support.

Given the high overlap in phenomenology between cardiac diseases and SSD, we aimed to explore PSS as a substantial component of SSD in a population of patients with cardiac disease. Previous research has indicated that subjective symptom burden is not directly associated to the severity of the disease itself^10^. Instead, psychological factors play a key role in the persistence of symptoms, which then contributes to maintaining the overall disease burden^36^. Research on individuals at risk for heart failure has demonstrated varying levels of symptom burden, independent of disease stage or risk, highlighting the importance of adopting a bio-psycho-social perspective^37^. A recent study by Müller-Tasch et al. (2024) also demonstrated that somatic symptoms are significantly associated with depressive comorbidity in patients with chronic heart failure^38^.

Löwe et al.^10^ stated that patients with PSS show frequent and potentially harmful overuse of healthcare. Our results showed a significantly higher number of visits to general practitioners as well as cardiologists for patients with PSS. Former studies showed an overuse of invasive cardiac procedures especially in Germany^39^. This study does not give an insight into whether the significantly higher healthcare utilization is appropriate, protective, or harmful. Considering the absence of significant differences in cardiac disease, comorbidities, or the number of medications as biomedical characteristics among patients with and without PSS, it would be valuable to investigate the direction and causality in future studies. These could analyze whether there is an association between invasive overuse and PSS in patients with cardiac disease. This knowledge could be helpful in efficiently guiding patients through the healthcare system. Konnopka et al. (2013) showed which costs are associated with somatic symptom severity in patients with medically unexplained symptoms^17^. Along the lines of these results, the costs due to PSS in patients with cardiac disease could be quantified.

### Limitations

Even though the study is exploratory it is important to note that the statistical power to detect predictors of PSS group affiliation and associations with healthcare utilization was only moderate. By defining the inclusion criteria to select patients with cardiac disease, we can ensure the focus on the target group of interest. However, given that this study involves secondary analysis, the sample exhibits high heterogeneity in terms of cardiac diseases. This diversity is valuable for an initial exploratory analysis aimed at defining PSS and enhancing our understanding of this patient group. Future studies should consider a larger sample size to test for replicability and should focus on a more differentiated approach of cardiac disease. This approach would allow for the identification of variations in characteristics, predictors, and specific needs among different subgroups within the population of patients with cardiac disease.

In this study, different approaches were used to define patients with and without PSS. Due to the study design of this secondary analysis, we were limited to utilizing longitudinal data spanning a 3-month period. Within criteria of related diagnoses such as the somatic symptom disorder according to the DSM-5, persistence is defined as six months or longer^2^. An extension of assessments over a longer period of time, such as six months, would improve our knowledge on the development of somatic symptoms in this patient group. Additionally, this study compares two approaches (psychometric-driven and data-driven) to define PSS in patients with cardiac disease in an explorative way. Further research should continue analyzing and specifying a definition of PSS in this patient group.

Finally, the study is based mainly on self-reporting. Biomedical factors, including cardiac disease, comorbidities, and medications, were assessed by screening the medical records of the patients and confirming the diagnoses during the cardiac consultation. Nevertheless, this study aimed to increase knowledge about factors characterizing and predicting PSS, which are based on subjective and individual perceptions^10^. The self-report questionnaires utilized were chosen as they have been shown to have reasonable psychometric properties in cardiology and primary care. In future research, social factors such as working circumstances and social interactions could be of interest against the background of the bio-psycho-social model^13^.

### Conclusion

To the best of our knowledge, this is the first study reporting on characteristics and predictors of patients with cardiac disease with PSS compared to those without PSS. Biomedical factors, except for angina pectoris as a cardiac factor, did not show significant differences in characteristics and prediction of patients with cardiac disease showing PSS. Additionally, psychological factors such as higher depression severity as well as sociodemographic factors such as higher age and female gender seemed to predict the development of PSS in this patient group. Due to these identified predictors, it could be advisable to evaluate PSS, especially in patients with cardiac disease at a higher age, female gender, significant angina pectoris, and elevated depression severity.

This longitudinal study provides insights into an initial exploration of defining PSS in patients with cardiac disease while describing its characteristics and predictors. PSS is one of the diagnostic criteria for somatic symptom disorder (SSD) as outlined in the DSM-5^2^. By expanding our knowledge about PSS in this patient group, we are also progressing towards the challenge of identifying somatic symptom disorder in patients with cardiac disease. It is noteworthy that approximately one-third of patients with cardiac disease exhibits PSS. Given the exploratory nature of this study, specific conclusions regarding changes in routine care cannot be drawn. However, suggestions for future research include focusing on patient-oriented treatment approaches or implementing screening measures, such as the assessment of depression severity, to enhance healthcare for patients with cardiac disease experiencing PSS and to support early identification. To comprehensively address the needs and appropriate healthcare, it is crucial to expand knowledge about PSS within this patient group. This can be achieved by analyzing patients with distinct cardiac diseases, other somatic diseases, or individuals at risk of developing cardiac diseases in future studies. Such research would enable the generalization and evaluation of initial findings observed in this longitudinal study.

## Electronic supplementary material

Below is the link to the electronic supplementary material.

Supplementary Material 1

## Data Availability

The data that supports the findings of this study is available on request from the corresponding author.

## Abbreviations

CCS: Canadian Cardiovascular Society
GAD-7: Generalized Anxiety Disorder Scale-7
PHQ-9: Patient Health Questionnaire-9
PSS: Persistent somatic symptoms
SSD: Somatic symptom disorder
SSS-8: Somatic Symptom Scale-8
