# Supplementary material for: Characteristics and predictors of persistent somatic symptoms in patients with cardiac disease
Source: Sci Rep. 2024 Oct 26;14:25517. doi: 10.1038/s41598-024-76554-z (PMC11513025; doi:10.1038/s41598-024-76554-z)

**Supplement A**

Dendrogram displaying hierarchical clustering results for group assignment.


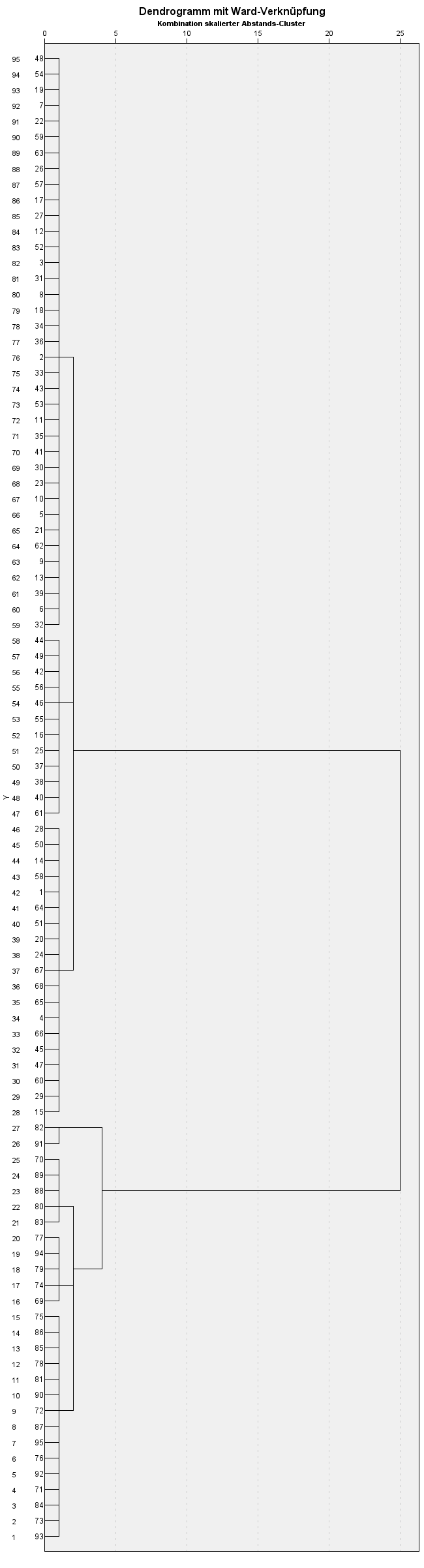


Cases

Squared euclidean distance

**Supplement B**

Elbow plot for determining optimal clusters using Ward’s method.


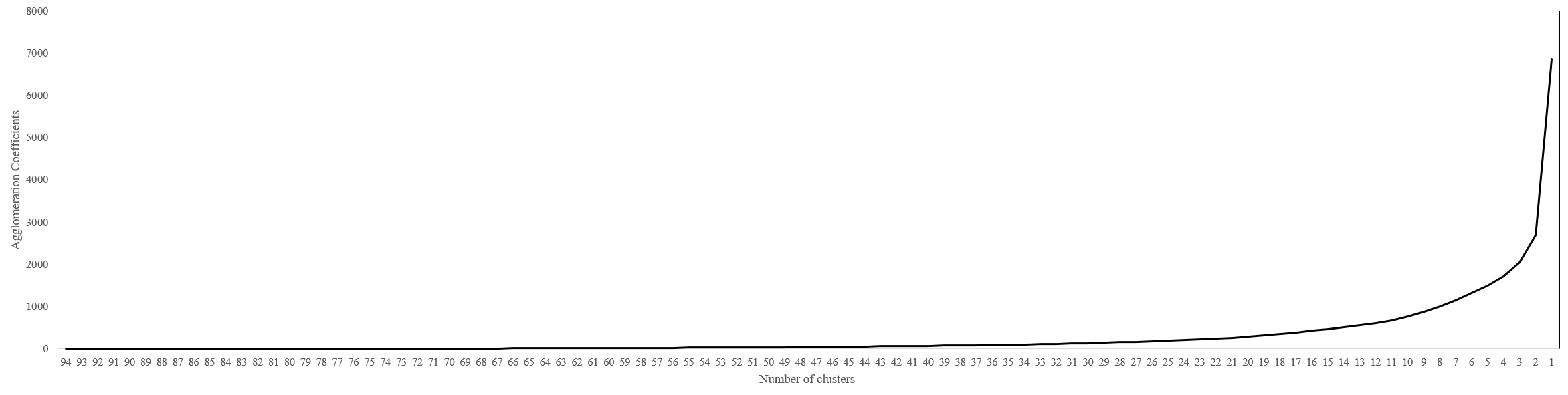

Supplement: Supplementary file 1 — Supplementary Material 1 [file 41598_2024_76554_MOESM1_ESM.docx]
